# Automated 4D analysis of dendritic spine morphology: applications to stimulus-induced spine remodeling and pharmacological rescue in a disease model

**DOI:** 10.1186/1756-6606-4-38

**Published:** 2011-10-07

**Authors:** Sharon A Swanger, Xiaodi Yao, Christina Gross, Gary J Bassell

**Affiliations:** 1 Department of Cell Biology, Emory University, 615 Michael St. NE, Atlanta, GA 30322, USA; 2 Department of Neurology, Emory University, 615 Michael St. NE, Atlanta, GA 30322, USA

**Keywords:** dendritic spine morphology, fragile X syndrome, automated image analysis, BDNF, dendritic spine remodeling, live cell imaging, 3D reconstruction, synapse, FMRP, PI3K inhibitor

## Abstract

Uncovering the mechanisms that regulate dendritic spine morphology has been limited, in part, by the lack of efficient and unbiased methods for analyzing spines. Here, we describe an automated 3D spine morphometry method and its application to spine remodeling in live neurons and spine abnormalities in a disease model. We anticipate that this approach will advance studies of synapse structure and function in brain development, plasticity, and disease.

## Background

Dendritic spines are dynamic, actin-rich protrusions that form the postsynaptic compartment at most glutamatergic synapses [1]. Synapse strength is closely correlated with dendritic spine morphology, and synaptic activity regulates spine number and shape during brain development, behavioral learning, and aging [2-4]. In addition, abnormal spine morphology is prevalent in neurological diseases such as intellectual disabilities, autism spectrum disorders, schizophrenia, mood disorders, and Alzheimer's disease [5-7]. Although many details regarding the spine structure-synapse function relationship remain unclear, it is evident that spine morphology can impact excitatory neurotransmission and is an important aspect of neuronal development, plasticity, and disease [6,8-10].

The lack of automated methods for quantifying spine number and geometry has hindered analysis of the mechanisms linking spine structure to synapse function [11]. Cultured neurons are the primary model system for studying the basic mechanisms regulating neuronal structure and function as these mechanistic studies require complex designs and large sample sizes in order to produce meaningful results. While several recent reports have described automated algorithms for analyzing neuron morphology *in vivo *[12-18], few independent studies have validated these methods [19,20] and there are no established methods for automated 3D spine analysis in cultured neurons. Son et al. developed an automated spine analysis algorithm using 2D images of cultured neurons, but 2D analyses do not consider a significant amount of information including all protrusions extending into the z-plane [21]. The majority of spine morphology studies have relied on manual measurements, which are time consuming, often biased by experimenter error and fatigue, and have limited reproducibility [14].

Here, we present, validate, and apply an automated 3D approach using the commercially available software program Filament Tracer (Imaris, Bitplane, Inc.). Filament Tracer has been used for automated spine detection *in vivo*, but geometric measurements were limited to spine head width [22,23]. Also, we have used Filament Tracer to facilitate spine density calculations in cultured neurons, but this analysis required manual validation and extensive editing of false-positive spines [24]. Now, our improved approach generates an accurate 3D reconstruction without any manual validation. Moreover, our approach can be applied to either fixed or live neurons as well as images acquired using either widefield fluorescence or confocal microscopy.

To demonstrate the applicability of our approach, we analyzed changes in spine morphology following acute brain-derived neurotrophic factor (BDNF) application in live hippocampal neurons. We verified our method by showing that acute BDNF treatment increased spine head volume, as was previously published [25]. Furthermore, we demonstrated that BDNF application induced rapid alterations in spine neck and length geometry and resulted in an overall maturation of the dendritic spine population within 60 minutes. We also applied our method to the study of aberrant spine morphology in a mouse model of fragile X syndrome (FXS), an inherited intellectual disability [26]. We not only accurately detected the established spine abnormalities in cultured neurons from this mouse model, but we also demonstrated that these abnormalities were rescued by inhibiting phosphoinositide-3 kinase activity, a potential therapeutic strategy for FXS [24]. These findings demonstrate that our approach is an efficient and accurate method for investigating dendritic spine development and plasticity as well as neurological disease mechanisms and therapies.

## Results and discussion

### Automated detection and 3D measurement of dendritic spines

**Figure 1:**
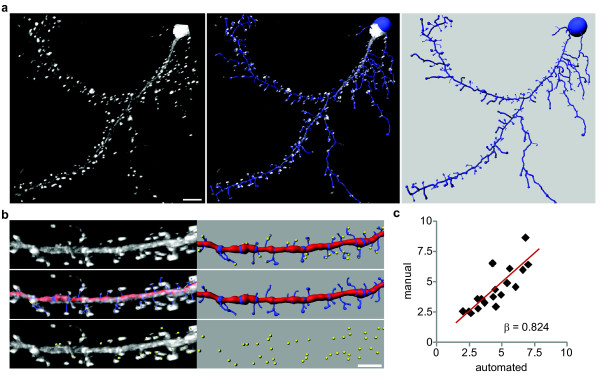
**Automated detection of dendritic spines using images of Lifeact-expressing neurons**. *(a) *Images depict a representative 3D reconstruction of a 17 *DIV *rat hippocampal neuron expressing Lifeact-ruby (white) and the automated trace generated by Filament Tracer (blue). Scale bar is 10 μm. *(b) *Automated and manual spine counts were performed within the same dendritic regions. Top: A dendritic segment of a Lifeact-ruby-expressing neuron (white) and the corresponding automated trace (dendrite: red, spines: blue) as well as manually marked spines (yellow spheres) are shown. Middle: Lifeact-ruby signal (white) is overlayed by the automated trace (shown alone at right). Bottom: Lifeact-ruby signal (white) is overlayed by manual spine marks (yellow; shown alone at right). *(c) *For each dendritic segment, the manual spine count was plotted against the automated count. Linear regression analysis showed that manual and automated spine detection were significantly correlated (n = 28; β = 0.824; p < 0.001).

The accurate study of dendritic spine morphology requires a method that incorporates effective neuron labeling with unbiased spine detection and measurement. To establish the most effective method for labeling and detecting spines in cultured hippocampal neurons, we tested several fluorescent markers including the lipophilic dye DiI and plasmids encoding soluble eGFP, membrane-tagged eGFP, and mRFPruby-tagged Lifeact, a small actin binding peptide [27]. The labeled neurons were fixed, and z-series images were acquired using a widefield fluorescence microscope. Following deconvolution, the images were analyzed with two different software programs: NeuronStudio, a program used for automated 3D neuron tracing *in vivo *[12], and Filament Tracer (Imaris, Bitplane, Inc.), a commercially available 3D tracing software. Universal parameters for accurate automated tracing of a large dataset could not be identified using NeuronStudio with any fluorescent label or using Filament Tracer with DiI-labeled or GFP-expressing neurons (data not shown). However, accurate 3D traces were automatically generated from images of Lifeact-ruby-expressing neurons (Figure 1a). While GFP is commonly used for morphological analyses, we found that generating accurate traces of GFP-expressing neurons required extensive manual editing of false-positive spines. Images of Lifeact-expressing neurons could be used to generate automated traces with universal parameters and no manual editing. Of note, Lifeact-expressing neurons have been previously shown to exhibit normal actin dynamics and dendritic spine morphology [27,28]. Consequently, we describe here the validation and application of an automated spine analysis method using Filament Tracer and images of Lifeact-expressing neurons.

To generate the 3D reconstructions for spine analysis, we selected a dendritic region that was 40 - 60 μm in length and void of dendritic branch points and crossing neurites. A point within the dendrite and at the edge of the selected region was assigned as the dendrite starting point, and the following parameters were set: *minimum dendrite end diameter *(0.75 μm; empirically determined to be the minimum dendritic width enabling accurate tracing), *minimum spine end diame*ter (0.215 μm; 2 times the pixel width), and *maximum spine len*gth (5 μm) [29]. The dendritic segment was then traced and volume rendered using automatic thresholds without any additional manual input or editing. On occasion the algorithm inappropriately assigned dendritic protrusions as dendrites instead of spines, so we applied a mathematical filter that selected all dendritic protrusions ≤ 5 μm in length and assigned them as spines. To validate the automated spine detection, spine density was calculated within the same dendritic regions using manual and automated analyses (Figure 1b). The automated measures accurately predicted the manual spine counts as determined by linear regression analysis (Figure 1c) [30]. The mean spine density (spines per 10 μm) did not significantly differ between the manual (4.36 ± 0.46) and automated (4.47 ± 0.41) analyses (Student's t-test, *P *= 0.836), but there was a consistent trend toward higher spine density using the automated method. The coefficient of variation was lower for the automated results (0.9) as compared to the manual measurements (0.11), suggesting that automated spine detection was slightly more reproducible than manual detection.

While spine number reflects the quantity of excitatory synapses, spine geometry is linked with excitatory synapse function and is also an important outcome measure in dendritic spine studies [10]. Spine head size is positively correlated with postsynaptic density (PSD) size, cell surface GluA receptor number, and synaptic vesicle content in the associated presynaptic terminal [31,32]. Spine length and neck width likely affect calcium signaling within spines as well as signaling from the spine to the dendrite shaft [33-35]. To evaluate how effectively our approach measured spine geometry, automated measurements of spine head width, neck width, and length were compared to manual measurements (Table 1). Unexpectedly, the distributions for each parameter significantly differed between the manual and automated methods (*N*_manual _= 411; *N*_auto _= 423 spines; Kolmogorov-Smirnov Test; head width: *D *= 0.394; neck width: *D *= 0.510; length: *D *= 0.178; all *P *< 0.001). Given these conflicting results, we evaluated the precision and accuracy of the automated and manual methods. To analyze precision, we evaluated specific characteristics of each dataset and found that the standard deviation and coefficient of variation were consistently smaller for the automated method (Table 1). Furthermore, the manual measurement distributions were more skewed than the automated distributions, indicating that the manual method yields distributions shifted further from the normal distribution as compared to automated analyses. Together, these data indicate that our automated approach is a more precise spine analysis method than manual measurements. To evaluate the accuracy of our approach, we used published ultrastructural data to estimate population statistics for spine head width, neck width, and length [10,29,31,36]. For each geometric parameter, the mean, median, and range values of the automated distributions (shown in Table 1) were more similar to the estimated population statistics (Table 2) than the manual values. For example, the estimated median head and neck widths garnered from several published ultrastructural studies were 0.40 μm and 0.15 μm, respectively. Our automatically determined median head and neck widths were 0.46 μm and 0.11 μm, respectively; whereas, our manually determined median head and neck widths were 0.60 μm and 0.23 μm, respectively. These data suggest that the automated approach generated data that was more accurate than manual measurements.

**Table 1:** Statistical comparison of geometric spine measurements

	Head width		Neck width		Length	
	
	Manual	Auto	Manual	Auto	Manual	Auto
**Neurons**						
Mean (μm)	0.67	0.40	0.27	0.17	1.91	1.73
SD	±0.22	±0.08	±0.07	±0.03	±0.73	±0.26
CV	0.32	0.20	0.27	0.20	0.38	0.15
**Spines**						
Median (μm)	0.60	0.44	0.23	0.11	1.50	1.33
Range (μm)	2.36	0.79	1.50	0.57	4.60	4.85
Skewness	1.25	0.10	2.66	2.09	1.03	0.84

The mean, standard deviation (SD), and coefficient of variation (CV) were calculated for the manual and automated measurements of average spine head width, neck width, and length per neuron (μm; *N *= 28 neurons). The median, range, and skewness were calculated for the distributions of spine head width, neck width, and length determined using the manual (*N *= 411 spines) and automated methods (*N = *423 spines) on the same 28 neurons.

**Table 2:** Estimated population statistics based on published electron microscopy studies

	Head width	Neck width	Length
Median	0.40	0.15	1.36
Mean	0.46	0.15	1.50
Range	0.84	0.42	4.80

Geometric spine measurements (μm) from previously published electron microscopy studies were pooled to generate estimated median, mean, and range values for the population of dendritic spines on hippocampal neurons [10,29,31,36,77].

In addition, dendritic spines were classified as stubby, mushroom, or thin using the aforementioned geometric measures; this is a widely used scheme to assess the proportions of mature and immature spines within a population [10,36,37]. While similar proportions of mushroom and thin spines were reported by both methods, the manual method reported a significantly lower proportion of stubby spines than the automated method (Table 3). On close examination, we observed that spines classified as stubby by the automated method were often manually classified as thin, due to an increased length measurement, or were manually determined to be a region of the dendrite shaft rather than a protrusion. In agreement with these observations, it is evident from the literature that manual spine analyses consistently underestimate the proportion of stubby spines and overestimate spine length at the low end of the distribution when compared to automated and semi-automated methods [12,17,38,39]. It is important to note that, given the resolution limit of light microscopy, some spine heads may not be distinguishable from short and wide spine necks. While electron microscopy affords the resolution to make such distinctions, light microscopy is a more versatile and practical approach for mechanistic studies of dendritic spine structure. Altogether, these results indicate that our method accurately and precisely reports spine number and geometry in cultured neurons. Moreover, our method is a significant advance over current spine analysis methods as dendritic spine detection and 3D measurements are entirely automated, thus greatly reducing the time burden and removing experimenter biases.

**Table 3:** Statistical comparison of spine shape classification

	Stubby		Mushroom		Thin	
	
	Manual	Auto	Manual	Auto	Manual	Auto
Median	7.0%	13.5%	65.5%	59.4%	11.7%	9.3%
SD	±11.4	±7.1	±20.0	±10.9	±15.5	±10.1
CV	1.10	0.49	0.33	0.18	0.99	0.80
*P*	0.033*		0.895		0.346	

Dendritic spines were classified as stubby, mushroom, or thin using the manually or automatically generated geometric measurements. *P*: The Kolmogorov Smirnov test was used to compare the manual and automated distributions (*significant difference between the manual and automated measures).

### Automated tracking of dendritic spines in live neurons

Dendritic spine density and morphology are dynamically regulated by many extracellular cues and neurotransmitters. For example, many more dendritic protrusions are formed during development than remain into adulthood, indicating that spine formation and morphogenesis are highly regulated processes; yet, the mechanisms determining which spines become stabilized remain unclear. In the adult brain, stimulus induced potentiation of the postsynaptic response can convert spines with small heads to large spines, whereas large spines can shrink in response to long-term depression of the postsynaptic response [40,41]. However, the detailed mechanisms governing these differential responses remain poorly understood. Therefore, time-lapse imaging in living neurons is an essential tool for studying stimulus-induced synapse development and plasticity.

**Figure 2:**
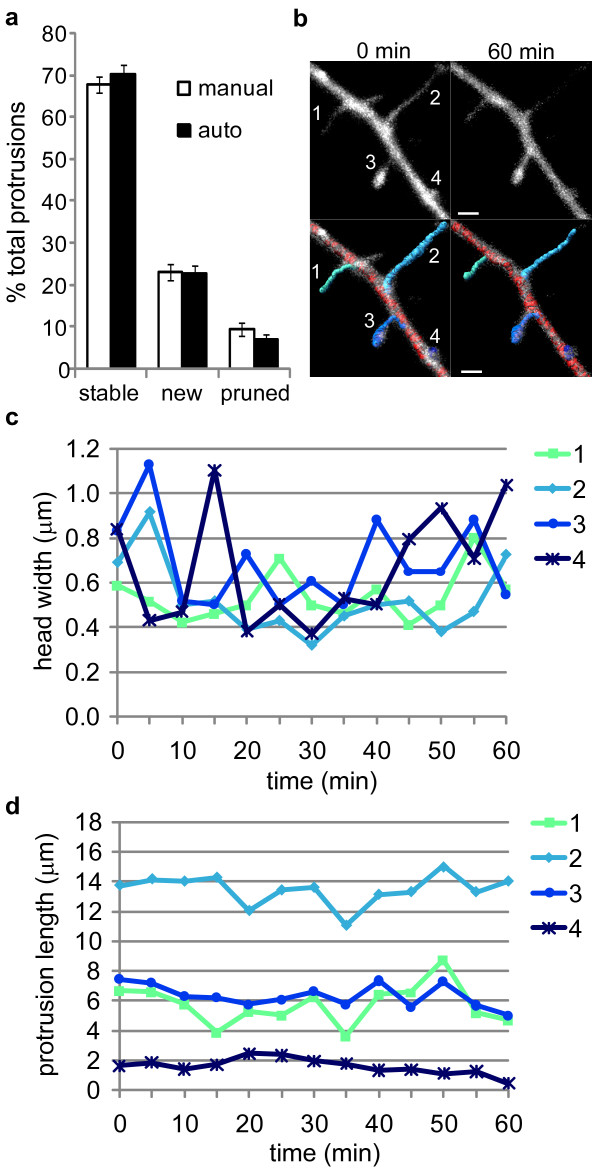
**Automated tracking of dendritic protrusions in live neurons**. 11 *DIV *hippocampal neurons were transfected with a vector expressing Lifeact-ruby, and 24 hrs later were imaged at 5 min intervals for 1 hr. *(a) *Individual protrusions were tracked across the time series manually and with our automated method. This histogram shows the percentages of stable, new, and pruned protrusions for both methods. *(b) *Images depict 12 *DIV *neurons expressing Lifeact-ruby (white; top) overlayed with automated 3D reconstructions (bottom). The dendrite shaft is red and each tracked protrusion is labeled 1 through 4 (shades of blue and green). Images from t = 0 and 60 min are shown, and the scale bar is 5 μm. *(c) *Head width and *(d) *protrusion length were plotted versus time for each protrusion; the labels 1 through 4 in the legend correspond to the labels 1 through 4 in panel (b).

To test how effectively our automated approach tracked dendritic protrusions in live hippocampal neurons, 12 *DIV *neurons expressing Lifeact-ruby were imaged at 5 min intervals for 1 hr. The 3D reconstructions were generated as described above with a few modifications. A dendrite starting point was defined for each time point using the AutoDepth mode in Imaris Filament Tracer. The automated trace was built using these existing dendrite start points and the following geometric parameters: *minimum dendrite end diameter *(0.75 μm), *minimum spine end diameter *(0.3 μm; empirically determined to be the minimum end diameter allowing accurate spine detection), and *maximum spine length *(15.0 μm). The *maximum spine length *was set at 15 μm to include dendritic filopodia, which are long and dynamic protrusions involved in spine and dendrite development [42,43]. Filopodia are included in this analysis because they are abundant on the 12 *DIV *neurons used for these experiments, whereas they are nearly absent on the mature neurons (17 *DIV*) used for the fixed neuron experiments described in Figure 1 and Table 1. The proportions of stable, new, and pruned dendritic protrusions (spines and filopodia) measured with the automated method were similar to those determined manually, suggesting that our automated approach allows detection and tracking of individual spines across time (Figure 2a). Moreover, we demonstrated that the morphology of individual spines can be tracked (Figure 2b) and quantified (Figure 2c and 2d) over time using our automated approach. Taken together, these data indicate that Lifeact-expressing neurons combined with this automated spine analysis approach is a valid method for the 4D tracking of dendritic protrusions in live neurons.

### Acute BDNF treatment induces synapse maturation through spine remodeling

**Figure 3:**
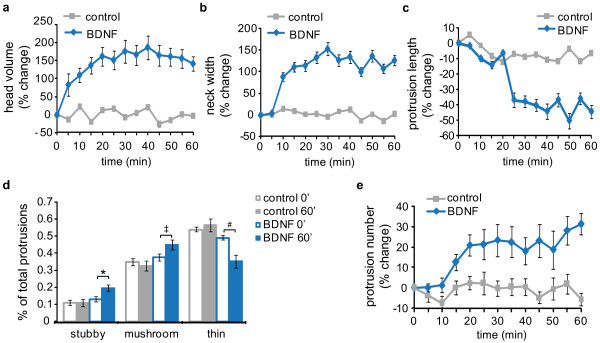
**Acute BDNF treatment induces maturation of the dendritic spine population**. 11 *DIV *hippocampal neurons were transfected with a vector expressing Lifeact-ruby, and 24 hrs later, the neurons were treated with vehicle or 100 ng/ml BDNF followed by time-lapse imaging every 5 minutes for 1 hr. Each protrusion was tracked across time and measured using the automated method. *(a) *Head volume was plotted as the percent change from the initial time point (T_0_). Statistical analyses were performed to compare protrusion head volume at T_0 _and T_60 _(*N *= 105 - 135 spines; Kruskal Wallis test with repeated measures; Control: *P *= 0.548; BDNF: *P *= 0.001). *(b) *Neck width was plotted and analyzed as above (Control: *P =*0.91; BDNF: *P = *0.0002). *(c) *Dendritic protrusion length was plotted and analyzed as above (Control: *P *= 0.648; BDNF: *P *= 0.017). *(d) *Dendritic protrusions were classified as stubby, mushroom, or thin at each time point. The percentages of total protrusions within each class are presented for T_0 _and T_60 _(*P = 0.038, ^‡^*P *= 0.044, ^#^*P = *0.015). *(e) *The number of protrusions within a dendritic region was determined using Imaris Filament Tracer and compared between T_0 _and T_60 _(*N *= 25 - 30 neurons; repeated measures ANOVA, post-hoc Tukey's test [Control: *P *= 0.219; BDNF: *P *= 0.014]).

To test the usefulness of our approach, we analyzed the acute effects of BDNF on spine morphology in live neurons (Figure 3a). BDNF is a neurotrophin that not only supports neuron differentiation and survival, but it is also an important regulator of synaptic signaling and plasticity [44]. The canonical mechanism for BDNF-induced synapse maturation is through chronic exposure and a transcription-dependent pathway [45]. However, BDNF also enhances glutamatergic neurotransmission through rapid, local signaling events [44], and recently Tanaka et al. showed that acute BDNF treatment increased dendritic spine head volume by ~150% within 25 minutes [25]. Here, we used cultured hippocampal neurons and our automated 4D approach to investigate the effects of acute BDNF application on dendritic spine morphology. Similar to the previous study, BDNF increased mean head volume by ~160% within 20 min, and this effect was maintained for 60 min (Figure 3a). In addition, we found that BDNF increased mean neck width by 125% (Figure 3b) and decreased mean protrusion length by 45% (Figure 3c). Spine classification analysis revealed significant increases in stubby and mushroom spine proportions and a decrease in the proportion of thin protrusions following BDNF treatment (Figure 3d). Finally, BDNF increased protrusion number by ~25% (Figure 3e). Together, these findings indicate that acute BDNF treatment leads to an overall maturation of the dendritic spine population in a manner consistent with enhanced synaptic efficacy.

In support of the above assertion, the observed increases in head and neck width and the decrease in protrusion length are associated with increased signaling between the dendritic spine and shaft, which promotes greater signal integration within the neuron [33-35]. Furthermore, we observed an increased proportion of mushroom-shaped spines, which have many GluA receptors and large PSDs; whereas, BDNF decreased the proportion of thin protrusions, which often lack surface GluA receptors and have less defined PSDs [46]. Importantly, our results agree with previous studies showing that acute BDNF enhances postsynaptic glutamate receptor function, increases excitatory postsynaptic currents, and increases intracellular calcium concentration in hippocampal neurons [44]. Thus, our observations provide extensive morphological evidence supporting a role for BDNF in the acute regulation of synapse structure.

**Figure 4:**
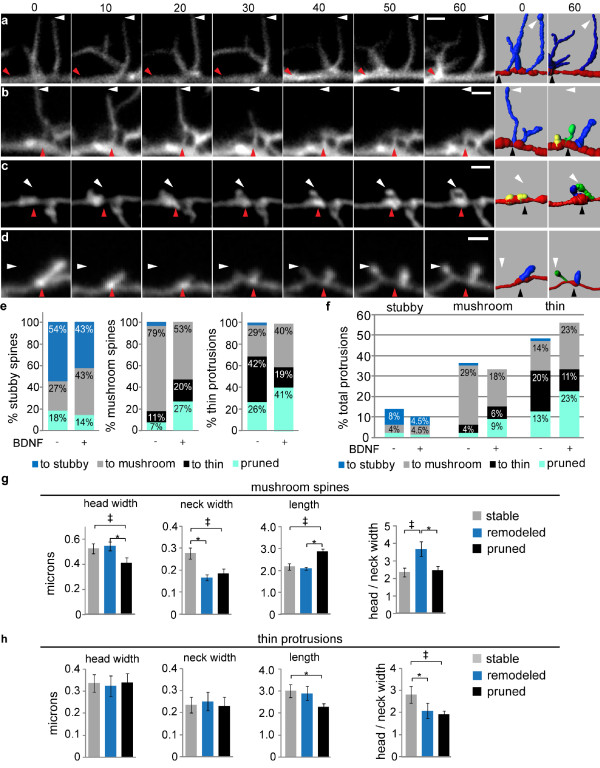
**Acute BDNF induces specific types of spine remodeling**. 12 *DIV *hippocampal neurons expressing Lifeact-ruby were treated with vehicle or 100 ng/ml BDNF and imaged at 5 min intervals for 1 hr. Each protrusion was classified as stubby, mushroom, or thin at t = 0 and 60 min. *(a-d) *At left, each each time series (0 - 60 min) depicts a representative type of dendritic protrusion remodeling observed during our analysis. At right, the automated 3D reconstructions illustrate the classification of each protrusion at t = 0 and 60 min (stubby: yellow, mushroom: green, thin: blue). *(e) *The diagram shows the percentages of pre-existing stubby, mushroom, and thin protrusions that were remodeled (to stubby, to mushroom, to thin) or pruned under control and BDNF-treated conditions. *(f) *Using the same dataset as in (e), we calculated the total incidence for each type of remodeling under control and BDNF-treated conditions. The histogram depicts the percentages of total protrusions that were initially stubby, mushroom, or thin and were either remodeled (to stubby, to mushroom, or to thin) or pruned. *(g) *The initial (t = 0) mean protrusion head width, neck width and length as well as the head width/neck width ratio were determined for mushrooms spines that were either stable (maintained mushroom morphology), remodeled into thin protrusions, or pruned within the 60 min imaging period following BDNF treatment (*N *= 24 - 32 spines; ANOVA, post-hoc Tukey's test; head width: ^‡^*P *= 0.032, *P = 0.017; neck width: *P = 0.020, ^‡^*P = *0.037; length: ^‡^*P *= 0.032, *P = 0.017; head/neck ratio: *P = 0.002, ^‡^*P = *0.001). *(h) *The group means listed above were determined for thin protrusions (t = 0) that were stable (maintained thin morphology), remodeled into mushroom spines, or pruned during 30 min. BDNF treatment (*N = *36 - 47 spines; ANOVA, post-hoc Tukey's test; length: *P = 0.002; head/neck ratio: *P = 0.037).

To determine how dendritic protrusions were remodeled to achieve the population effects described above, we tracked individual protrusions across time and quantified their morphogenesis. In this analysis, we asked three basic questions regarding remodeling: 1) does initial protrusion morphology affect remodeling, 2) what are the incidences of specific types of remodeling, and 3) what, if any, geometric parameters are associated with specific changes in morphology? We also evaluated whether BDNF treatment impacted these aspects of protrusion dynamics. Qualitatively, we observed several types of spine and filopodia remodeling such as: transient and highly dynamic thin protrusions, the morphogenesis of long, thin protrusions into mushroom-shaped spines, the growth of stubby-spines into mushroom-shaped spines, and de novo mushroom spine formation (Figure 4a-d and sample movie in Additional File 1).

To quantitatively analyze remodeling, we calculated the percentages of each protrusion type (stubby, mushroom, or thin) that maintained classification, remodeled into another protrusion type, or were pruned over 60 min. All newly formed spines were excluded from this analysis. Under control conditions, similar proportions of thin protrusions and stubby spines were either remodeled into mushroom spines (28.9% and 27.3%, respectively) or pruned (26.3% and 18.2%, respectively), whereas 78.6% of mushroom spines maintained their shape and only 7.1% were pruned (Figure 4a). Acute BDNF treatment increased the remodeling of both thin and stubby protrusions into mushroom spines (40.5% and 42.9%, respectively) as well as the percentage of mushroom spines (26.7%) and thin protrusions (40.5%) that were pruned. However, BDNF slightly decreased the percentage of stubby spines that were pruned (14.3%). Interestingly, thin- and mushroom-shaped protrusions rarely morphed into stubby spines, and stubby spines were never observed to remodel into thin protrusions. These observations suggest that stubby and thin protrusions have similar propensities for remodeling into mushroom spines, but they likely do so through distinct mechanisms.

Among the total spine population, thin protrusions had the highest incidence of remodeling, and mushroom spines showed the lowest incidence of remodeling (Figure 4b). Following BDNF treatment, stubby spines had the lowest incidence of remodeling (see sample movie in Additional File 2), suggesting that stubby spines may not be simply a transitional structure, but that they might have an important end function as a stable structure under certain conditions. The BDNF-induced increases in the proportion and stability of stubby spines, reported in Figure 3, are difficult to interpret, because the role of stubby spines in neuronal function remains controversial. Stubby spines do not maintain or recruit GluA receptors as efficiently as mushroom spines, nor do they form synapses as often [47-50]. On the other hand, stubby spines might have enhanced coupling to the dendritic shaft as compared to the other spine types [51]. Also, stubby spine incidence is increased during learning *in vivo*, and it has been theorized that they are transitional structures that will be enlarged/stabilized or have undergone shrinkage due to synaptic weakening [2,6,41,46,52,53]. Our data suggest that it is unlikely for an increase in stubby spines to result from the weakening of mushroom spines or the retraction of thin protrusions, but it is possible that the increase in stubby spines is linked to the increase in total protrusion number following BDNF stimulation. Future studies in systems having a higher overall incidence of stubby spines, perhaps neurons in an earlier developmental stage, will be important for advancing our understanding stubby spine formation, remodeling, and function.

These results also have implications regarding spine formation. Several mechanisms have been proposed for how stable, mushroom-shaped spines are formed, including growth of mushroom spines from the dendritic shaft, morphogenesis of a filopodia into a mushroom spine, and retraction of filopodia into the dendritic shaft resulting in a shaft or stubby spine synapse followed by growth of a mushroom spine at the same location [47,54-56]. Our data clearly support the formation of mushroom spines de novo and through morphogenesis of an existing filopodia (Figure 4) as has been previously observed *in vitro *and *in vivo *[53,55-63]. However, our data suggest that mushroom spine formation via filopodia retraction into a stubby spine followed by re-growth is not a common occurrence, at least in this model system, as we rarely observed morphogenesis of a filopodia into a stubby spine. Whether filopodia were retracted fully into the shaft and re-emerged as mushroom spines at the same locus was not evaluated in the current study, but this analysis is possible using our automated method and can be investigated in future studies.

To investigate whether any geometric parameters were associated with BDNF-induced remodeling, the initial (t = 0) mean head width, neck width, and protrusion length were compared among stable, remodeled, and pruned mushroom spines or stable, remodeled, and pruned thin protrusions (Figure 4g,h). Large neck width was the best predictor of mushroom spine stability, whereas head width was not significantly different between stable and remodeled mushroom spines (Figure 4g). Mushroom spine pruning was associated with reduced head and neck width and increased length compared to the other two groups (Figure 4g). For thin protrusions, a high ratio of head width to neck width was the best indicator of stability (see Figure 4d and the sample movie in Additional File 3). Interestingly, these data are consistent with functional studies reporting that large neck width is associated with greater synaptic strength [33] and synaptic potentiation of thin protrusions is promoted by maintaining high concentrations of signaling molecules within the head, which might be due to a high ratio of head width to neck width [46,64].

An interesting observation was that BDNF decreased the percentage of mushroom spines that remained as mushroom spines from 79% to 53% (Figure 4e). Moreover, 26% of mushroom spines were pruned following BDNF. Both observations imply BDNF-induced turnover of mushroom spines, suggesting that the overall net gain in spine maturation (increased density and spine width, reduced length) (Figure 3) involves extensive remodeling. This process may involve pruning of mushroom spines that passed certain thresholds approaching immature phenotypes (e.g. low head or neck width, or increased length), which are apparently replaced by more mature mushroom spines developed from other less mature populations (thin, stubby).

In the future, it will be important to study the different mechanisms underlying specific types of spine formation and remodeling, such as the distinctions between stubby and thin protrusion remodeling into mushroom spines. Furthermore, there is still much debate regarding the functional significance of different spine morphologies in brain development, plasticity, and disease. One necessary step towards understanding the structure-function relationship of dendritic spines is generating reproducible and interpretable spine morphology data. The accuracy and speed of our method makes it well-suited for studies of this type, and we anticipate that our approach will facilitate studies on spine structure and its relation to synapse function.

In addition to advancing morphological studies, the described technique has the potential to facilitate studies evaluating the synaptic localization of specific molecules. The fluorescence intensity of multiple channels can be automatically quantified within each dendritic spine; thus, one could evaluate whether a particular fluorescently tagged or stained molecule is differentially localized between spine types or shows altered localization following a pharmacological, molecular, or genetic manipulation. Therefore, the combination our optimized spine analysis method with automated quantification of spine fluorescence creates a powerful and efficient technique for simultaneously studying spine morphology and the molecules regulating synapse structure and function.

### Inhibiting PI3 kinase activity rescues dendritic spine defects in neurons from Fmr1 KO mice

The importance of dendritic spine morphology is emphasized by the fact that spine abnormalities are associated with varied neurological diseases such as intellectual disabilities, neurodegenerative diseases, and psychiatric disorders [5]. Cultured neurons are a valuable model system for studying the mechanisms underlying brain diseases; as such, it is critical that spine analysis methods effectively detect aberrant spine phenotypes in disease models and identify treatments that ameliorate disease phenotypes. Here, we used our approach to study spine morphology in neurons from *Fmr1 *knockout mice, a mouse model of fragile X syndrome (FXS).

**Figure 5:**
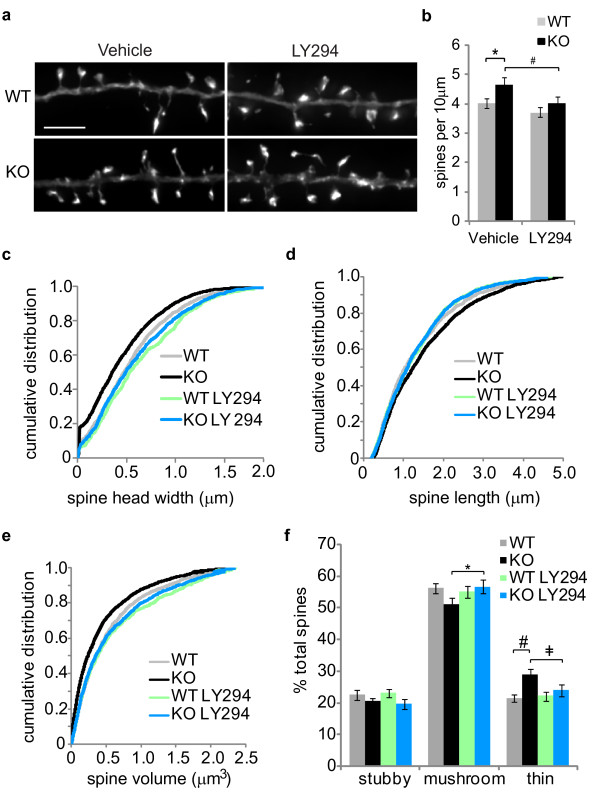
**A PI3 kinase inhibitor rescues spine morphology in neurons from *Fmr1 *knockout mice**. *(a) *Hippocampal neurons cultured from wild type (WT) or *Fmr1 *knockout (KO) mice were treated with vehicle or a PI3 kinase inhibitor (LY294, 10 μM) for 72 hours starting at 15 *DIV*. Neurons were transfected with a plasmid encoding Lifeact-ruby at 17 *DIV *and fixed 24 hours later. The images depict representative dendritic regions from deconvolved z-series images (scale bar is 5 μm). *(b) *Spine density was measured using our automated approach for WT and KO neurons treated with either vehicle or LY294 (n = 55-60 neurons; ANOVA [*F *= 3.996, *P = *0.009]; post-hoc Fisher's LSD: *P = 0.017, ^#^*P *= 0.028). Cumulative distributions of *(c) *spine head width, *(d) *length, and *(e) *volume were plotted for each group (Kolmogorov-Smirnov test: head width [WT vs. KO: *P = *0.002, WT vs. WT LY294: *P = *0.235, KO vs. KO LY294: *P = *0.009], spine length [WT vs. KO: *P = *0.009, WT vs. WT LY294: *P = *0.537, KO vs. KO LY294: *P = *0.014], spine volume [WT vs. KO: *P *< 0.001, WT vs. WT LY294: *P = *0.158, KO vs. KO LY294: *P *< 0.001]). *(f) *Spines were classified as stubby, mushroom, and thin based on the automated geometric measurement, and the values were plotted as percentage of total spines per treatment group (n = 55 - 60 neurons; ANOVA with post-hoc Fisher's LSD: *P = 0.043, #*P *= 0.006, ^‡^*P = *0.043).

FXS is an inherited intellectual disability caused by the loss of fragile X mental retardation protein (FMRP), an RNA binding protein that regulates mRNA transport and local protein synthesis at synapses [26]. Patients with FXS display increased dendritic spine density, an increased incidence of thin spines, and increased mean spine length, and these abnormalities are recapitulated in neurons from *Fmr1 *knockout mice [65-68]. In a previous study, we detected increased spine density in cultured hippocampal neurons from *Fmr1 *knockout mice using a semi-automated spine analysis method in which the experimenter manually edited an automated trace; however, we were unable to detect any other defects in spine morphology with this method [[27], and unpublished observations]. Using our fully automated method, we accurately detected the established spine phenotypes in 18 *DIV *hippocampal neurons from *Fmr1 *knockout mice: increased spine density, decreased spine head width, increased spine length, and decreased spine volume (Figure 5). Furthermore, there were less mushroom-shaped spines and more thin spines in FMRP-deficient neurons, which is in line with previous reports [66,69-71]. These data further demonstrate the validity of our approach as well as its usefulness for studying neurological diseases.

Next, we investigated whether treating hippocampal neurons with a phosphoinositide-3-kinase (PI3K) inhibitor affected spine morphology. Previously, we discovered that inhibiting PI3K activity is a potential therapeutic strategy for FXS. We showed that the loss of FMRP leads to excess PI3K activity and treatment with a PI3K inhibitor, LY294002, rescues several neuronal phenotypes in *Fmr1 *knockout mice, including aberrant synaptic protein synthesis, GluA receptor internalization, and dendritic spine density [24]. Here, using our automated approach, we reproduced our previous findings by demonstrating that LY294002 treatment (10 μM for 72 hrs) reduced spine density in hippocampal neurons from *Fmr1 *knockout mice to wild type levels (Figure 5b). Furthermore, our analysis revealed that LY294002 treatment significantly increased spine head width, decreased spine length, and increased spine volume in neurons from *Fmr1 *KO mice (Figure 5c-e). Additionally, LY294002 significantly increased mushroom-shaped spines and decreased thin spines in FMRP-deficient neurons such that all spine proportions were similar to those of wild type neurons (Figure 5f). These data indicate that inhibiting PI3K activity not only rescues increased spine density in a mouse model of FXS, but also restores aberrant spine shape to the wild type morphology. These findings are an important advance of our previous findings and further support the pharmacological inhibition of PI3K as a potential FXS treatment strategy [72]. More broadly, these data demonstrate that our automated approach can be used to study dendritic spine abnormalities and potential pharmacotherapeutics in neurological disorders.

Although spine defects are apparent in many brain diseases, a vital unanswered question is whether altered spine morphology contributes to disease onset and progression or is secondary to disordered neuronal activity [5,73]. Of note, cortical neurons in a mouse model of Alzheimer's disease exhibit reduced spine density, a phenotype evident in patients with Alzheimer's disease, but these neurons do not show overt electrophysiological impairments; whereas, other mouse models of Alzheimer's disease show both structural and functional phenotypes in cortical neurons [74]. In addition, it is possible to alter synaptic efficacy without inducing long-term changes in spine morphology, and altering spine morphology through manipulating the neuronal cytoskeleton is not always sufficient to alter synapse function [4]. These data highlight the complexity inherent in the spine structure-synapse function relationship and emphasize the importance of developing powerful techniques for studying the mechanisms regulating spine morphology in brain development, plasticity, and disease.

## Conclusions

We have developed an automated 3D approach for dendritic spine analysis using neurons expressing fluorescently labeled Lifeact. This versatile method can be applied to images of either fixed or live cultured neurons that were collected using widefield fluorescence or confocal microscopy. The increased speed and accuracy of our automated spine analysis, as compared to manual spine assessments, is critical for uncovering the complicated mechanisms underlying normal and aberrant dendritic spine formation and remodeling. Using our automated approach, we showed that acute BDNF treatment leads to rapid spine remodeling consistent with enhanced synaptic efficacy. We also found that inhibiting PI3 kinase activity rescues aberrant spine shape in neurons from a mouse model of FXS. We predict that this method will significantly advance studies of glutamatergic synapse structure and function in neuronal health and disease.

## Methods

### Neuron culture, transfection, and drug treatments

Hippocampal neurons were isolated from embryos at E18 (rat) or E17 (mouse) and cultured at high-density as previously described with minor modifications [75]. Rat hippocampal neurons were cultured in Neurobasal medium (Invitrogen) supplemented with NS21 [76]. Neurons were either plated on 15 mm glass coverslips and co-cultured with glia, or plated on 35 mm MatTek glass bottom dishes in glia-conditioned media that was exchanged every 2 days with new glia-conditioned media.

Fixed neuron experiments: 16-17 *DIV *neurons were transfected with plasmids encoding Lifeact-ruby (a generous gift from Dr. Roland Wedlich-Soldner, Max Planck Institute, Martinsried, Germany), Lifeact-GFP, GFP, or membrane-tagged GFP using NeuroMag (OZBiosciences). DiI labeling was performed on 16 *DIV *neurons by incubating the coverslips covered with a small volume of neuronal culture media containing Vybrant DiI solution (Invitrogen) for 25 min at 37°C. For LY294002 experiments, 15 *DIV *neurons were treated with 10 μM LY294002 or an equivalent volume of DMSO for 72 hrs total; the culture media was exchanged with conditioned media containing freshly prepared drug (or vehicle) after 24 and 48 hrs.

Live neuron experiments: 11 *DIV *rat hippocampal neurons were transfected with a plasmid encoding Lifeact-ruby using Lipofectamine 2000 and used for imaging 24 hrs later. Thirty minutes prior to imaging, neurons were equilibrated to glia-conditioned imaging media (phenol red-free Neurobasal media supplemented with HEPES, sodium pyruvate, NS21, and Glutamax). For BDNF experiments: One hour prior to imaging, neurons were starved in glia-conditioned imaging media without NS21, and immediately prior to time lapse imaging neurons were treated with BDNF (100 ng/ml; Peprotech) or vehicle (H_2_O).

### Microscopy

Widefield fluorescence: Twenty-four hours after transfection, hippocampal neurons were fixed with 4% paraformaldehyde in 1x phosphate-buffered saline (PBS), washed 3 times with 1x PBS, and the coverslips were mounted on microscope slides with propyl gallate-containing polyvinyl alcohol. Neurons were imaged on a Nikon Eclipse Ti microscope with a Nikon Intensilight and Photometrics Coolsnap HQ2 camera. GFP was imaged using a 480/40 excitation filter, a 535/50 emission filter, and a 505 dichroic (Nikon), and ruby and DiI were imaged using a 545/30 excitation filter, a 620/60 emission filter, and a 570 dichroic. Images were acquired using a 60X oil-immersion objective (Nikon Plan Apo, N.A. 1.40). Z-series images were acquired at 0.15 μm increments through the entire visible dendrite.

Confocal laser scanning: Time lapse imaging was performed on a Nikon A1R confocal encased in a plexiglass humidified chamber maintained at 37°C and 5% CO_2 _using a 60X oil immersion objective (Nikon Plan Apo, N.A. 1.40). Images of Lifeact-ruby were collected using a 561 nm laser for excitation and a 650 emission filter. Z-series were acquired at 0.15 μm increments, and a Nikon Perfect Focus system was enabled for the duration of the experiment.

### Image processing

Images were deconvolved in AutoQuant X (MediaCybernetics) using the blind algorithm, which employs an iteratively refined theoretical PSF. No further processing was performed prior to image analysis. For preparation of figures, maximum intensity Z-projections were created in Imaris (Figures 1 and 5) or average intensity Z-projections were created using ImageJ (Figures 2 and 4). For visualization, brightness and contrast levels were adjusted using ImageJ.

### Automated image analysis

In Imaris Surpass mode, a new filament was created using the Autopath mode and a region of interest (ROI) was selected. To select an ROI, we identified a dendritic region 40 - 60 μm length that was distal to a dendritic branch point and void of crossing neurites or any additional dendritic branch points. A *minimum dendrite end diameter *of 0.75 μm was entered and a single dendrite starting point was assigned at the edge of the ROI. For time-lapse image series, a single dendrite starting point was assigned at each timepoint by using the AutoDepth mode. Automatic thresholds were used for assigning dendrite end points and dendrite surface rendering. To trace spines, the *maximum spine length *and *minimum spine end diameter *were set at 5 μm and 0.215 μm, respectively, for fixed neuron experiments and 15 μm and 0.3 μm, respectively, for live imaging experiments. Automatic thresholds were used for generating spine seed points and surface rendering. After generating the trace, a filter was applied to ensure all dendritic protrusions ≤ 5 μm (or 15 μm) were assigned as spines; to do so, we created a filter that selected all dendritic segments with "Branch level" = 2 and "length" ≤ 5 (or 15) and the selected segments were assigned as spines by choosing "Assign as spine" under the *Edit *tab. All of the geometric parameters and filters were set, or loaded from a previously analyzed image, at the start of the analysis session after which the software maintained these values. For each subsequent image processed, an ROI was selected, a dendrite starting point was assigned, and then the trace was built by clicking "Finish". To apply the filter, the *Filter *tab was opened (which automatically selected the appropriate segments), then by clicking on the *Edit *tab followed by "Assign as spine" the final 3D trace was generated. Filament statistics were exported into Excel (Microsoft), where they were compiled and graphed.

### Manual image analysis

Manual analyses were performed in Imaris Surpass mode using the same dendritic ROIs as above. The dendrite length was measured using Measurement Points and each spine was marked using Spots (Imaris). Using Measurement Points, head width was measured at the maximum width of the spine tip, neck width was measured at the minimum point along the spine length, and spine length was measured from the dendrite shaft to the spine tip. Each ROI was processed in duplicate and the values were averaged.

### Spine classifications

Spines were classified into groups termed stubby, mushroom, and thin. These groups were established as follows: stubby (length ≤1 μm and neck width/head width < 1.5), mushroom (neck width/head width ≥ 1.5 and length ≤5 μm), and thin (1 < length ≤ 5 μm and neck width/head width < 1.5) [36]. Classification for both manual and Filament Tracer, were computed in Excel using the following formulas:

$$\begin{array}{lll} \text{Stubby}: & =F\left(AND\left(length\leq 5,\;head∕neck\leq 1.5\right),1,0\right)\; & \text{(1)}\\ \text{Mushroom}: & =IF\left(AND\left(length\leq 5,\;head∕neck\geq 1.5\right),1,0\right)\; & \text{(2)}\\ \text{Thin}: & =IF\left(AND\left(length\leq 5,\;length>1,\;head∕neck\leq 1.5\;\right),1,0\right)\; & \text{(3)}\\ & \; & \text{(4)} \end{array}$$

These logic statements return a value of 1 if true and 0 if false. The total number of spines in each class was tallied by summing the results of the logic statements. For live imaging experiments, a maximum length of 15 μm was used instead of 5 μm.

### Statistics

Unless otherwise noted, statistics were completed using PASW Statistics 18 (SPSS, Inc). All datasets were analyzed for equal variance using Levene's test and normality using the Kolmogorov Smirnov test. Normally distributed datasets were compared using either Student's t-test or an ANOVA followed by post-hoc tests as noted in figure legends. Non-normal datasets were compared using the Mann-Whitney U test or Kruskal Wallis test. Cumulative distributions were compared using the Kolmogorov-Smirnov test. Alpha was set at 0.05 for all comparisons. Power analysis was performed using G*Power 3.1.2 (University of Kiel, Germany) with β = 0.8 and α = 0.05, and effect size and standard deviation were determined using pilot experiment results. The experimenter was blind to treatment and genotype during all image analysis.

## Competing interests

CG and GJB declare that they are inventors on patent application PCT/US2010/055387.

## Authors' contributions

SAS contributed to the conception and design of the study, tested and validated the method, performed and analyzed the live imaging experiments, performed the statistical analyses, and drafted the manuscript. XY performed the mouse neuron culture, transfection, and imaging for the LY294002 experiments. CG participated in the design and analysis of the LY294002 experiments. GJB contributed to the conception, analysis, and coordination of the study, and edited the manuscript. All authors have read and approved the final manuscript.

## Supplementary Material

Additional file 1 **Individual dendritic spines undergo remodeling that can be tracked across time**. A 12 *DIV *hippocampal neuron expressing Lifeact-ruby was treated with BDNF (100 ng/ml) and image at 5 in intervals for 1 hr. In this representative neuron, a thin protrusion undergoes extensive remodeling (blue) and a newly formed spine emerges from the dendritic shaft (red) and morphs into a mushroom-shaped spine. Click here for file

Additional file 2 **Stubby spines are highly stable following BDNF stimulation**. A 12 *DIV *hippocampal neuron expressing Lifeact-ruby was treated with BDNF (100 ng/ml) and imaged at 5 min intervals for 1 hr. The stubby spine (center) remains stable for the duration of the experiment; whereas, neighboring thin protrusions can be seen extending and retracting. Click here for file

Additional file 3 **Thin protrusions with a high head/neck width ratio remain stable**. A 12 *DIV *hippocampal neuron expressing Lifeact-ruby was treated with 100 ng/ml BDNF and imaged at 5 min intervals for 1 hr. The long protrusion with a defined head that is much wider than the neck (at right) remains stable throughout the experiment; whereas, neighboring protrusions, which do not exhibit increased width at the tip, undergo dynamic structural changes and/or are pruned. Click here for file

## References

[B1] Edwards FA Anatomy and electrophysiology of fast central synapses lead to a structural model for long-term potentiation Physiol Rev 1995 75 759 787 7480162 10.1152/physrev.1995.75.4.759

[B2] Holtmaat A Svoboda K Experience-dependent structural synaptic plasticity in the mammalian brain Nat Rev Neurosci 2009 10 647 658 10.1038/nrn2699 19693029

[B3] Kasai H Fukuda M Watanabe S Hayashi-Takagi A Noguchi J Structural dynamics of dendritic spines in memory and cognition Trends Neurosci 2010 33 121 129 10.1016/j.tins.2010.01.001 20138375

[B4] Alvarez VA Sabatini BL Anatomical and physiological plasticity of dendritic spines Annu Rev Neurosci 2007 30 79 97 10.1146/annurev.neuro.30.051606.094222 17280523

[B5] Penzes P Cahill ME Jones KA Vanleeuwen JE Woolfrey KM Dendritic spine pathology in neuropsychiatric disorders Nat Neurosci 2011 14 285 293 10.1038/nn.2741 21346746 PMC3530413

[B6] Lee KJ Kim H Rhyu IJ The roles of dendritic spine shapes in Purkinje cells Cerebellum 2005 4 97 104 10.1080/14734220510007842 16035191

[B7] Glantz LA Lewis DA Dendritic spine density in schizophrenia and depression Arch Gen Psychiatry 2001 58 203 10.1001/archpsyc.58.2.203 11177126

[B8] Hayashi Y Majewska AK Dendritic spine geometry: functional implication and regulation Neuron 2005 46 529 532 10.1016/j.neuron.2005.05.006 15944122

[B9] Bourne JN Harris KM Coordination of size and number of excitatory and inhibitory synapses results in a balanced structural plasticity along mature hippocampal CA1 dendrites during LTP Hippocampus 2010 10.1002/hipo.20768 PMC2891364 20101601

[B10] Bourne JN Harris KM Balancing structure and function at hippocampal dendritic spines Annu Rev Neurosci 2008 31 47 67 10.1146/annurev.neuro.31.060407.125646 18284372 PMC2561948

[B11] Svoboda K The past, present, and future of single neuron reconstruction Neuroinformatics 2011 9 97 98 10.1007/s12021-011-9097-y 21279476

[B12] Rodriguez A Ehlenberger DB Dickstein DL Hof PR Wearne SL Automated three-dimensional detection and shape classification of dendritic spines from fluorescence microscopy images PLoS One 2008 3 e1997 10.1371/journal.pone.0001997 18431482 PMC2292261

[B13] Rodriguez A Ehlenberger DB Hof PR Wearne SL Rayburst sampling, an algorithm for automated three-dimensional shape analysis from laser scanning microscopy images Nature protocols 2006 1 2152 2161 10.1038/nprot.2006.313 17487207

[B14] Wearne SL Rodriguez A Ehlenberger DB Rocher AB Henderson SC Hof PR New techniques for imaging, digitization and analysis of three-dimensional neural morphology on multiple scales Neuroscience 2005 136 661 680 10.1016/j.neuroscience.2005.05.053 16344143

[B15] Janoos F Mosaliganti K Xu X Machiraju R Huang K Wong ST Robust 3D reconstruction and identification of dendritic spines from optical microscopy imaging Med Image Anal 2009 13 167 179 10.1016/j.media.2008.06.019 18819835 PMC2663851

[B16] Zhang Y Chen K Baron M Teylan MA Kim Y Song Z Greengard P Wong ST A neurocomputational method for fully automated 3D dendritic spine detection and segmentation of medium-sized spiny neurons Neuroimage 2010 50 1472 1484 10.1016/j.neuroimage.2010.01.048 20100579 PMC2839064

[B17] Zhang Y Zhou X Witt RM Sabatini BL Adjeroh D Wong ST Dendritic spine detection using curvilinear structure detector and LDA classifier Neuroimage 2007 36 346 360 10.1016/j.neuroimage.2007.02.044 17448688

[B18] Mukai H Hatanaka Y Mitsuhashi K Hojo Y Komatsuzaki Y Sato R Murakami G Kimoto T Kawato S Automated Analysis of Spines from Confocal Laser Microscopy Images: Application to the Discrimination of Androgen and Estrogen Effects on Spinogenesis Cerebral cortex 2011 10.1093/cercor/bhr059 PMC3209797 21527787

[B19] Bloss EB Janssen WG Ohm DT Yuk FJ Wadsworth S Saardi KM McEwen BS Morrison JH Evidence for reduced experience-dependent dendritic spine plasticity in the aging prefrontal cortex J Neurosci 2011 31 7831 7839 10.1523/JNEUROSCI.0839-11.2011 21613496 PMC3398699

[B20] Scotto-Lomassese S Nissant A Mota T Neant-Fery M Oostra BA Greer CA Lledo PM Trembleau A Caille I Fragile X mental retardation protein regulates new neuron differentiation in the adult olfactory bulb J Neurosci 2011 31 2205 2215 10.1523/JNEUROSCI.5514-10.2011 21307257 PMC3682409

[B21] Stevens JK Trogadis J Reconstructive three-dimensional electron microscopy. A routine biologic tool Anal Quant Cytol Histol 1986 8 102 107 3730086

[B22] Shen H Sesack SR Toda S Kalivas PW Automated quantification of dendritic spine density and spine head diameter in medium spiny neurons of the nucleus accumbens Brain Struct Funct 2008 213 149 157 10.1007/s00429-008-0184-2 18535839

[B23] Staffend NA Loftus CM Meisel RL Estradiol reduces dendritic spine density in the ventral striatum of female Syrian hamsters Brain Struct Funct 2011 215 187 194 10.1007/s00429-010-0284-7 20953625 PMC3057377

[B24] Gross C Nakamoto M Yao X Chan CB Yim SY Ye K Warren ST Bassell GJ Excess phosphoinositide 3-kinase subunit synthesis and activity as a novel therapeutic target in fragile X syndrome J Neurosci 2010 30 10624 10638 10.1523/JNEUROSCI.0402-10.2010 20702695 PMC2924772

[B25] Tanaka J Horiike Y Matsuzaki M Miyazaki T Ellis-Davies GC Kasai H Protein synthesis and neurotrophin-dependent structural plasticity of single dendritic spines Science 2008 319 1683 1687 10.1126/science.1152864 18309046 PMC4218863

[B26] Bassell GJ Warren ST Fragile X syndrome: loss of local mRNA regulation alters synaptic development and function Neuron 2008 60 201 214 10.1016/j.neuron.2008.10.004 18957214 PMC3691995

[B27] Riedl J Crevenna AH Kessenbrock K Yu JH Neukirchen D Bista M Bradke F Jenne D Holak TA Werb Z Lifeact: a versatile marker to visualize F-actin Nat Methods 2008 5 605 607 10.1038/nmeth.1220 18536722 PMC2814344

[B28] Riedl J Flynn KC Raducanu A Gartner F Beck G Bosl M Bradke F Massberg S Aszodi A Sixt M Wedlich-Soldner R Lifeact mice for studying F-actin dynamics Nat Methods 2010 7 168 169 10.1038/nmeth0310-168 20195247

[B29] Papa M Bundman MC Greenberger V Segal M Morphological analysis of dendritic spine development in primary cultures of hippocampal neurons J Neurosci 1995 15 1 11 7823120 10.1523/JNEUROSCI.15-01-00001.1995 PMC6578316

[B30] Ludbrook J Linear regression analysis for comparing two measurers or methods of measurement: but which regression? Clin Exp Pharmacol Physiol 2010 37 692 699 10.1111/j.1440-1681.2010.05376.x 20337658

[B31] Harris KM Stevens JK Dendritic spines of CA 1 pyramidal cells in the rat hippocampus: serial electron microscopy with reference to their biophysical characteristics J Neurosci 1989 9 2982 2997 2769375 10.1523/JNEUROSCI.09-08-02982.1989 PMC6569708

[B32] Kasai H Matsuzaki M Noguchi J Yasumatsu N Nakahara H Structure-stability-function relationships of dendritic spines Trends Neurosci 2003 26 360 368 10.1016/S0166-2236(03)00162-0 12850432

[B33] Noguchi J Matsuzaki M Ellis-Davies GC Kasai H Spine-neck geometry determines NMDA receptor-dependent Ca2+ signaling in dendrites Neuron 2005 46 609 622 10.1016/j.neuron.2005.03.015 15944129 PMC4151245

[B34] Korkotian E Holcman D Segal M Dynamic regulation of spine-dendrite coupling in cultured hippocampal neurons Eur J Neurosci 2004 20 2649 2663 10.1111/j.1460-9568.2004.03691.x 15548208

[B35] Biess A Korkotian E Holcman D Diffusion in a dendritic spine: the role of geometry Phys Rev E Stat Nonlin Soft Matter Phys 2007 76 021922 17930080 10.1103/PhysRevE.76.021922

[B36] Harris KM Jensen FE Tsao B Three-dimensional structure of dendritic spines and synapses in rat hippocampus (CA1) at postnatal day 15 and adult ages: implications for the maturation of synaptic physiology and long-term potentiation J Neurosci 1992 12 2685 2705 1613552 10.1523/JNEUROSCI.12-07-02685.1992 PMC6575840

[B37] Peters A Kaiserman-Abramof IR The small pyramidal neuron of the rat cerebral cortex. The perikaryon, dendrites and spines Am J Anat 1970 127 321 355 10.1002/aja.1001270402 4985058

[B38] Fan J Zhou X Dy JG Zhang Y Wong ST An automated pipeline for dendrite spine detection and tracking of 3D optical microscopy neuron images of in vivo mouse models Neuroinformatics 2009 7 113 130 10.1007/s12021-009-9047-0 19434521 PMC2872186

[B39] Koh IY Lindquist WB Zito K Nimchinsky EA Svoboda K An image analysis algorithm for dendritic spines Neural Comput 2002 14 1283 1310 10.1162/089976602753712945 12020447

[B40] Matsuzaki M Honkura N Ellis-Davies GC Kasai H Structural basis of long-term potentiation in single dendritic spines Nature 2004 429 761 766 10.1038/nature02617 15190253 PMC4158816

[B41] Zhou Q Homma KJ Poo MM Shrinkage of dendritic spines associated with long-term depression of hippocampal synapses Neuron 2004 44 749 757 10.1016/j.neuron.2004.11.011 15572107

[B42] Yoshihara Y De Roo M Muller D Dendritic spine formation and stabilization Current opinion in neurobiology 2009 19 146 153 10.1016/j.conb.2009.05.013 19523814

[B43] Heiman MG Shaham S Twigs into branches: how a filopodium becomes a dendrite Current opinion in neurobiology 2010 20 86 91 10.1016/j.conb.2009.10.016 19939665 PMC2827671

[B44] Gottmann K Mittmann T Lessmann V BDNF signaling in the formation, maturation and plasticity of glutamatergic and GABAergic synapses Exp Brain Res 2009 199 203 234 10.1007/s00221-009-1994-z 19777221

[B45] Chapleau CA Larimore JL Theibert A Pozzo-Miller L Modulation of dendritic spine development and plasticity by BDNF and vesicular trafficking: fundamental roles in neurodevelopmental disorders associated with mental retardation and autism J Neurodev Disord 2009 1 185 196 10.1007/s11689-009-9027-6 19966931 PMC2788955

[B46] Bourne J Harris KM Do thin spines learn to be mushroom spines that remember? Curr Opin Neurobiol 2007 17 381 386 10.1016/j.conb.2007.04.009 17498943

[B47] Fiala JC Feinberg M Popov V Harris KM Synaptogenesis via dendritic filopodia in developing hippocampal area CA1 J Neurosci 1998 18 8900 8911 9786995 10.1523/JNEUROSCI.18-21-08900.1998 PMC6793554

[B48] Matsuo N Reijmers L Mayford M Spine-type-specific recruitment of newly synthesized AMPA receptors with learning Science 2008 319 1104 1107 10.1126/science.1149967 18292343 PMC2692967

[B49] Ashby MC Maier SR Nishimune A Henley JM Lateral diffusion drives constitutive exchange of AMPA receptors at dendritic spines and is regulated by spine morphology J Neurosci 2006 26 7046 7055 10.1523/JNEUROSCI.1235-06.2006 16807334 PMC6673929

[B50] Harris KM Structure, development, and plasticity of dendritic spines Curr Opin Neurobiol 1999 9 343 348 10.1016/S0959-4388(99)80050-6 10395574

[B51] Richardson RJ Blundon JA Bayazitov IT Zakharenko SS Connectivity patterns revealed by mapping of active inputs on dendrites of thalamorecipient neurons in the auditory cortex J Neurosci 2009 29 6406 6417 10.1523/JNEUROSCI.0258-09.2009 19458212 PMC2729683

[B52] Holtmaat AJ Trachtenberg JT Wilbrecht L Shepherd GM Zhang X Knott GW Svoboda K Transient and persistent dendritic spines in the neocortex in vivo Neuron 2005 45 279 291 10.1016/j.neuron.2005.01.003 15664179

[B53] Zuo Y Lin A Chang P Gan WB Development of long-term dendritic spine stability in diverse regions of cerebral cortex Neuron 2005 46 181 189 10.1016/j.neuron.2005.04.001 15848798

[B54] Yuste R Bonhoeffer T Genesis of dendritic spines: insights from ultrastructural and imaging studies Nature reviews Neuroscience 2004 5 24 34 10.1038/nrn1300 14708001

[B55] Papa M Segal M Morphological plasticity in dendritic spines of cultured hippocampal neurons Neuroscience 1996 71 1005 1011 10.1016/0306-4522(95)00490-4 8684603

[B56] Ziv NE Smith SJ Evidence for a role of dendritic filopodia in synaptogenesis and spine formation Neuron 1996 17 91 102 10.1016/S0896-6273(00)80283-4 8755481

[B57] Marrs GS Green SH Dailey ME Rapid formation and remodeling of postsynaptic densities in developing dendrites Nat Neurosci 2001 4 1006 1013 10.1038/nn717 11574832

[B58] Okabe S Miwa A Okado H Spine formation and correlated assembly of presynaptic and postsynaptic molecules J Neurosci 2001 21 6105 6114 11487634 10.1523/JNEUROSCI.21-16-06105.2001 PMC6763142

[B59] Knott GW Holtmaat A Wilbrecht L Welker E Svoboda K Spine growth precedes synapse formation in the adult neocortex in vivo Nat Neurosci 2006 9 1117 1124 10.1038/nn1747 16892056

[B60] Nagerl UV Kostinger G Anderson JC Martin KA Bonhoeffer T Protracted synaptogenesis after activity-dependent spinogenesis in hippocampal neurons J Neurosci 2007 27 8149 8156 10.1523/JNEUROSCI.0511-07.2007 17652605 PMC6672732

[B61] Kwon HB Sabatini BL Glutamate induces de novo growth of functional spines in developing cortex Nature 2011 474 100 104 10.1038/nature09986 21552280 PMC3107907

[B62] Konur S Yuste R Imaging the motility of dendritic protrusions and axon terminals: roles in axon sampling and synaptic competition Molecular and cellular neurosciences 2004 27 427 440 10.1016/j.mcn.2004.07.005 15555921

[B63] Lohmann C Finski A Bonhoeffer T Local calcium transients regulate the spontaneous motility of dendritic filopodia Nat Neurosci 2005 8 305 312 10.1038/nn1406 15711541

[B64] Grunditz A Holbro N Tian L Zuo Y Oertner TG Spine neck plasticity controls postsynaptic calcium signals through electrical compartmentalization J Neurosci 2008 28 13457 13466 10.1523/JNEUROSCI.2702-08.2008 19074019 PMC6671740

[B65] Irwin SA Patel B Idupulapati M Harris JB Crisostomo RA Larsen BP Kooy F Willems PJ Cras P Kozlowski PB Abnormal dendritic spine characteristics in the temporal and visual cortices of patients with fragile-X syndrome: a quantitative examination Am J Med Genet 2001 98 161 167 10.1002/1096-8628(20010115)98:2<161::AID-AJMG1025>3.0.CO;2-B 11223852

[B66] Irwin SA Idupulapati M Gilbert ME Harris JB Chakravarti AB Rogers EJ Crisostomo RA Larsen BP Mehta A Alcantara CJ Dendritic spine and dendritic field characteristics of layer V pyramidal neurons in the visual cortex of fragile-X knockout mice Am J Med Genet 2002 111 140 146 10.1002/ajmg.10500 12210340

[B67] Antar LN Li C Zhang H Carroll RC Bassell GJ Local functions for FMRP in axon growth cone motility and activity-dependent regulation of filopodia and spine synapses Molecular and cellular neurosciences 2006 32 37 48 10.1016/j.mcn.2006.02.001 16631377

[B68] Cruz-Martin A Crespo M Portera-Cailliau C Delayed stabilization of dendritic spines in fragile X mice J Neurosci 2010 30 7793 7803 10.1523/JNEUROSCI.0577-10.2010 20534828 PMC2903441

[B69] Bilousova TV Dansie L Ngo M Aye J Charles JR Ethell DW Ethell IM Minocycline promotes dendritic spine maturation and improves behavioural performance in the fragile X mouse model J Med Genet 2009 46 94 102 18835858 10.1136/jmg.2008.061796

[B70] Galvez R Greenough WT Sequence of abnormal dendritic spine development in primary somatosensory cortex of a mouse model of the fragile X mental retardation syndrome American journal of medical genetics Part A 2005 135 155 160 15880753 10.1002/ajmg.a.30709

[B71] de Vrij FM Levenga J van der Linde HC Koekkoek SK De Zeeuw CI Nelson DL Oostra BA Willemsen R Rescue of behavioral phenotype and neuronal protrusion morphology in Fmr1 KO mice Neurobiology of disease 2008 31 127 132 10.1016/j.nbd.2008.04.002 18571098 PMC2481236

[B72] Gross C Berry-Kravis EM Bassell GJ Therapeutic Strategies in Fragile X Syndrome: Dysregulated mGluR Signaling and Beyond Neuropsychopharmacology 2011 10.1038/npp.2011.137 PMC3238060 21796106

[B73] Portera-Cailliau C Which Comes First in Fragile X Syndrome, Dendritic Spine Dysgenesis or Defects in Circuit Plasticity? Neuroscientist 2011 10.1177/1073858410395322 21551076

[B74] Rocher AB Kinson MS Luebke JI Significant structural but not physiological changes in cortical neurons of 12-month-old Tg2576 mice Neurobiology of disease 2008 32 309 318 10.1016/j.nbd.2008.07.014 18721884 PMC2683422

[B75] Tiruchinapalli DM Oleynikov Y Kelic S Shenoy SM Hartley A Stanton PK Singer RH Bassell GJ Activity-dependent trafficking and dynamic localization of zipcode binding protein 1 and beta-actin mRNA in dendrites and spines of hippocampal neurons J Neurosci 2003 23 3251 3261 12716932 10.1523/JNEUROSCI.23-08-03251.2003 PMC6742306

[B76] Chen Y Stevens B Chang J Milbrandt J Barres BA Hell JW NS21: re-defined and modified supplement B27 for neuronal cultures J Neurosci Methods 2008 171 239 247 10.1016/j.jneumeth.2008.03.013 18471889 PMC2678682

[B77] Sorra KE Harris KM Overview on the structure, composition, function, development, and plasticity of hippocampal dendritic spines Hippocampus 2000 10 501 511 10.1002/1098-1063(2000)10:5<501::AID-HIPO1>3.0.CO;2-T 11075821

